# Natural products protect against spinal cord injury by inhibiting ferroptosis: a literature review

**DOI:** 10.3389/fphar.2025.1557133

**Published:** 2025-04-03

**Authors:** Wei She, Junxiao Su, Wenji Ma, Guohai Ma, Jianfu Li, Hui Zhang, Cheng Qiu, Xingyong Li

**Affiliations:** ^1^ School of Traditional Chinese and Western Medicine, Gansu University of Chinese Medicine, Lanzhou, Gansu, China; ^2^ Department of Orthopaedic Surgery, Gansu Provincial Hospital, Lanzhou, Gansu, China; ^3^ Department of Orthopaedic Surgery, The First Hospital of Lanzhou University, Lanzhou, Gansu, China; ^4^ Department of Orthopaedic Surgery, Qilu Hospital of Shandong University, Jinan, Shandong, China; ^5^ Department of Orthopedic Surgery, Peking Union Medical College Hospital, Peking Union Medical College and Chinese Academy of Medical Sciences, Beijing, China

**Keywords:** spinal cord injury, ferroptosis, natural product, GPx4, ROS

## Abstract

Spinal cord injury (SCI) is a severe traumatic condition that frequently results in various neurological disabilities, including significant sensory, motor, and autonomic dysfunctions. Ferroptosis, a recently identified non-apoptotic form of cell death, is characterized by the accumulation of reactive oxygen species (ROS), intracellular iron overload, and lipid peroxidation, ultimately culminating in cell death. Recent studies have demonstrated that ferroptosis plays a critical role in the pathophysiology of SCI, contributing significantly to neural cell demise. Three key cellular enzymatic antioxidants such as glutathione peroxidase 4 (GPX4), ferroptosis suppressor protein 1 (FSP1), and dihydroorotate dehydrogenase (DHODH), have been elucidated as crucial components in the defense against ferroptosis. Natural products, which are bioactive compounds mostly derived from plants, have garnered considerable attention for their potential therapeutic effects. Numerous studies have reported that several natural products can effectively mitigate neural cell death and alleviate SCI symptoms. This review summarizes fifteen natural products containing (−)-Epigallocatechin-3-gallate (EGCG), Proanthocyanidin, Carnosic acid, Astragaloside IV, Trehalose, 8-gingerol, Quercetin, Resveratrol, Albiflorin, Alpha-tocopherol, Celastrol, Hispolon, Dendrobium Nobile Polysaccharide, Silibinin, and Tetramethylpyrazine that have shown promise in treating SCI by inhibiting ferroptosis. Additionally, this review provides an overview of the mechanisms involved in these studies and proposes several perspectives to guide future research directions.

## Introduction

The spinal cord, situated within the vertebral canal, plays a critical role in connecting the cerebrum to peripheral neurons. Both sensory and motor functions of the extremities are contingent upon the integrity of the spinal cord. However, spinal cord injury (SCI) frequently results in various disabilities due to neurological impairments and is predominantly caused by traumatic events (Stanners et al., 2024). Patients with SCI often experience diverse neurological deficits, including localized loss of movement or sensation, paralysis below the injury level, or even life-threatening conditions in severe cases. Traumatic injuries, such as those caused by vehicular accidents, falls, and sports-related incidents, account for over 90% of SCIs (Alizadeh et al., 2019). According to previous studies, SCI not only severely impacts an individual’s quality of life but also imposes a substantial socioeconomic burden globally (GTBIaSCI Collaborators, 2019; Soendergaard et al., 2022; Fan et al., 2022). Currently, clinical treatments for SCI remain limited, with patient recovery primarily dependent on surgical decompression and pharmacological interventions (Sousa et al., 2025). Further research from multiple perspectives may facilitate neural restoration and repair.

Ferroptosis is a novel, non-apoptotic form of inducible cell death characterized by uncontrolled lipid peroxidation, iron accumulation, and dysregulated redox homeostasis (Dixon et al., 2012). Small molecules such as Erastin and RSL3, primarily designed to induce cell death in various types of tumors, are considered the canonical inducers of ferroptosis (Yang et al., 2014). Coined a decade ago, ferroptosis has been extensively studied worldwide and has been demonstrated in both human physiological and pathological processes (Jiang et al., 2021; Chen et al., 2021). Its role spans tumor progression, neuronal loss in Alzheimer’s or Parkinson’s, hepatic injury in nonalcoholic fatty liver disease (NAFLD), and myocardial damage in ischemia-reperfusion injury, highlighting its therapeutic potential as both an inducer and inhibitor (Jiang et al., 2021; Berndt et al., 2024; Dai et al., 2024). For the elimination of cancer cells, exogenous induction of ferroptosis through these inducers is feasible. However, in the context of neurodegenerative diseases, inhibiting ferroptosis can help prevent neuronal degeneration.

Natural products are a category of extractive molecules, derivatives, or leachates mostly derived from plants in nature (Rajesh and Sangeetha, 2024; Wang et al., 2024a). One of the most well-known classical herbal extracts is artemisinin, recognized globally as an effective antimalarial drug (Luo et al., 2024). Since this significant milestone, an increasing number of natural products have been studied for their potential in treating various diseases. However, there remains a lack of comprehensive summaries regarding these studies in the context of spinal cord injury (SCI). This review compiles and synthesizes related research on the use of natural products for treating SCI, particularly through the prevention of ferroptosis. Additionally, we propose several perspectives aimed at guiding future research directions.

## Mechanisms of ferroptosis

### Lipid peroxidation

Lipids encompass fatty acids, glycerides, steroids, phospholipids, and sphingolipids, which serve as fundamental components of cellular structure. They also play crucial roles in energy storage, energy provision, and signal transduction (Liu et al., 2020). Lipid peroxidation refers to the oxidative reaction in which unsaturated fatty acids participate to form peroxidized lipids. Low levels of lipid peroxidation are typically regulated and contribute to normal physiological processes (Ursini and Maiorino, 2020). However, when lipid peroxidation exceeds a certain threshold, it can lead to a loss of control and trigger ferroptosis (Barayeu et al., 2023). Consequently, lipid peroxidation levels are closely linked to ferroptosis. Cellular membranes, including those of intracellular organelles, are the most common targets of oxidative damage during ferroptosis. The generation of polyunsaturated fatty acids (PUFAs) from these membranes is fundamental to the induction of ferroptosis (Kagan et al., 2017). It is known that acyl-CoA synthetase long-chain family member 4 (ACSL4) promotes lipid peroxidation upstream, and its inhibition can reduce sensitivity to ferroptosis (Doll et al., 2017; Yuan et al., 2016). Currently, three types of enzymes-arachidonate lipoxygenases (ALOXs), cytochrome P450, and cyclooxygenase (PTGS)-have been reported to be involved in the regulation of lipid peroxidation (Yang et al., 2016; Chu et al., 2019; Zou et al., 2020; Li et al., 2017).

### Iron accumulation

Cellular iron metabolism is crucial for the process of ferroptosis. Iron, an essential trace element for the human body, plays a significant role in both health and disease (Berndt et al., 2024; Fang et al., 2023a). The intestine, kidneys, liver, and macrophages are key players in maintaining systemic iron balance (Bayır et al., 2023). Dietary iron is primarily absorbed into the bloodstream by intestinal epithelial cells in the form of ferric iron (Fe^3+^) and subsequently transferred into the cytosol via the transferrin receptor (TFRC) (Masaldan et al., 2018). Ferrous iron (Fe^2+^) is vital for oxygen transport, energy metabolism, and the production of iron-sulfur proteins (Tang et al., 2021). TFRC-bound Fe^3+^ is reduced to Fe^2+^, and the solute carrier family 11 member 2 (SLC11A2/DMT1) facilitates its release (Song et al., 2021). Both iron-storage proteins, ferritin light chain (FTL) and ferritin heavy chain 1 (FTH1), can be degraded by lysosomes to increase free iron levels, thereby initiating ferroptosis (Tang et al., 2021). Furthermore, the iron-efflux protein solute carrier family 40 member 1 (SLC40A1), also known as ferroportin 1 (FPN), expels iron into the extracellular space, thereby preventing excessive iron accumulation and promoting ferroptosis (Song et al., 2016). Iron overload primarily induces the production of lipid reactive oxygen species (ROS) through the Fenton reaction during ferroptosis (Li et al., 2021). Hydroxyl radicals generated from the reaction of iron with hydrogen peroxide further interact with lipids to form lipid peroxide free radicals (Rice-Evans and Burdon, 1993). Additionally, iron disrupts redox homeostasis and promotes ROS accumulation, leading to an oxidative stress response that induces ferroptosis (Mancardi et al., 2021).

### Antioxidant system

**FIGURE 1:**
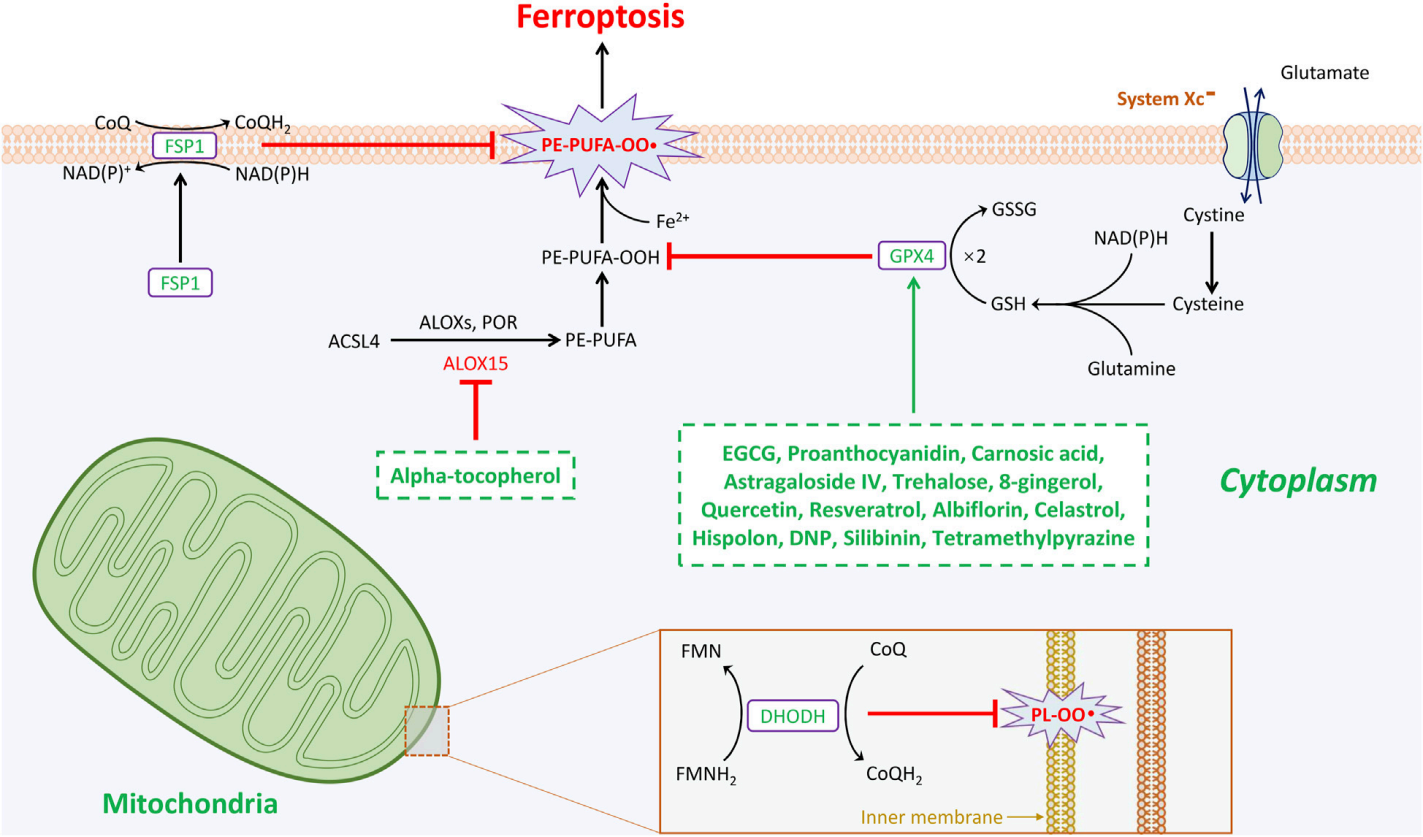
Natural products mediated anti-oxidative stress enzymatic systems in ferroptosis during the treatment of spinal cord injury. Ferroptosis is under control of GPX4-, FSP1-, and DHODH-dependent systems. GPX4 is the most important gatekeeper for ferroptosis and bolstered through the sustainment of GSH and cystine transportation of system Xc-activation. Cells are resistant to GPX4 inhibition through activating FSP1/CoQ10 system in cytoplasm to escape from ferroptosis. This GPX4-independent manner plays critical role in mitigating cellular ferroptosis. The third anti-oxidant system that DHODH-mediated ferroptosis protection in mitochondria is revealed. In the inner membrane of mitochondria, DHODH suppresses ferroptosis via the conversion of ubiquinone to ubiquinol that fights against oxidative damage on the phospholipid membrane. Total three gatekeepers presumably serve as potential targets for the treatment of spinal cord injury. Intriguingly, except alpha-tocopherol mitigates ferroptosis by targeting arachidonic acid 15-lipoxygenase (ALOX15), additional fourteen natural products inhibit ferroptosis all through GPX4-dependent pathway.

Numerous endogenous molecules have been identified as playing protective roles for cells in the fight against ferroptosis. Ferroptosis is closely associated with oxidative stress and the generation of reactive oxygen species (ROS) (Xie et al., 2021). Currently, three well-known cellular enzymatic antioxidants-glutathione peroxidase 4 (GPX4) (Yang et al., 2014; Friedmann et al., 2014), ferroptosis suppressor protein 1 (FSP1) (Bersuker et al., 2019; Doll et al., 2019), and dihydroorotate dehydrogenase (DHODH) (Mao et al., 2021), have been elucidated as being involved in ferroptosis defense (Figure 1). Moreover, glutathione S-transferase-Z1 (GSTZ1) (Wang et al., 2021), mitochondrial Superoxide Dismutase 2 (SOD2) (Liu et al., 2021a), thioredoxin-domain-containing 12 (TXNDC12) (Tang et al., 2023), thioredoxin reductase 1 (TXNRD1) (Liu et al., 2021b), nitric oxide synthase 2 (NOS2, also known as inducible nitric oxide synthase) (Kapralov et al., 2020), microsomal glutathione S-transferase 1 (MGST1) (Kuang et al., 2021), phospholipase A2 group VI (PLA2G6) (Sun et al., 2021), peroxiredoxins (PRDX) (Lovatt et al., 2020), 7-Dehydrocholesterol (7-DHC) (Li et al., 2024a; Freitas et al., 2024), and GTP cyclohydrolase 1 (GCH1) (Kraft et al., 2020) have all been reported as antioxidants that inhibit ferroptosis. Although the functions of these gatekeepers arise from distinct mechanisms, they all play critical roles in preventing cell death due to ferroptosis (Qiu et al., 2022).

GPX4 is an antioxidant defense enzyme that plays a crucial role in ferroptosis by scavenging lipid oxidative reactive oxygen species (ROS) (Ingold et al., 2018). Notably, GPX4 is widely regarded as a key inhibitory point for anti-ferroptosis (Yang et al., 2014). The canonical induction of ferroptosis significantly depends on the inactivation of the GPX4-mediated thiol system. The stable reduction of the oxidative form of GPX4 necessitates the continuous biosynthesis of glutathione (GSH) from cysteine (Shimada et al., 2016; Carlson et al., 2016). Cellular cysteine is derived from cystine, which is transported from the extracellular environment by a well-known membrane transporter called system Xc-, also known as xCT. This transporter consists of two subunits: solute carrier family 7 member 11 (SLC7A11) and SLC3A2 (Stockwell and Jiang, 2020). It is well recognized that Erastin directly targets SLC7A11, disrupting the transport of glutamine and cystine, thereby inducing ferroptosis through the obstruction of GSH biosynthesis and subsequent GPX4 inactivation (Dixon et al., 2012). In contrast, the classical induction of ferroptosis by RSL3 directly impacts GPX4 (Yang et al., 2014). Furthermore, the activation of antioxidant signaling pathways, such as the nuclear factor erythroid 2-related factor 2 (Nrf2), is instrumental in regulating GPX4 expression (Liu et al., 2024a; Shin et al., 2018). Increasing evidence has also revealed that several drugs, such as sulfasalazine and sorafenib, which induce ferroptosis, target GPX4 (Xie et al., 2016; Louandre et al., 2013). In the context of tumor or cancer eradication through ferroptosis, focusing on the inhibition of GPX4, the primary regulator of ferroptosis, is currently the preferred strategy.

The identification of FSP1 offers promising prospects for combating tumor cells (Zheng and Conrad, 2020). The designation of FSP1 is derived from apoptosis-inducing factor mitochondrial 2 (AIFM2), which has been previously reported to induce apoptosis (Wu et al., 2002). Despite the expression of ACSL4 and the inhibition of GPX4, tumor cells continue to exhibit resistance to ferroptosis, indicating the presence of alternative resistance mechanisms (Bersuker et al., 2019; Doll et al., 2019). Gene screening reveals a discrepancy in the expression of AIFM2 following the loss of GPX4. Moreover, tumor cells that overexpress AIFM2 provide significant protection against both pharmacological and genetic induction of ferroptosis. Following the demonstration of FSP1, further mechanisms indicate that cytosolic FSP1 translocates to the membrane after myristoylation, where it inhibits lipid peroxidation and ferroptosis. Additionally, both the N-myristoylation signal and the flavoprotein oxidoreductase domain of FSP1 are critical for its anti-ferroptotic functions. The mechanisms by which FSP1, in conjunction with NAD(P)H, converge with extra-mitochondrial ubiquinone (CoQ_10_) at the plasma membrane allow for the neutralization of lipid hydroperoxides, thereby countering ferroptosis. Intriguingly, FSP1 has also been validated as a participant in the noncanonical vitamin K cycle and in promoting lipid radical scavenging (Mishima et al., 2022). The phase separation of FSP1 induced by a class of compounds known as 3-phenylquinazolinones (represented by icFSP1) promotes ferroptosis, suggesting that FSP1 inhibition could serve as an effective anti-cancer therapy (Nakamura et al., 2023).

DHODH is an iron-dependent flavin mitochondrial enzyme that regulates *de novo* pyrimidine biosynthesis. Under normal inner mitochondrial metabolism, DHODH functions as an oxidoreductase, reducing ubiquinone (CoQ) to ubiquinol (CoQH_2_) in a non-GSH-dependent manner. This process generates a radical-trapping antioxidant that mitigates reactive oxygen species (ROS) (Mao et al., 2021). In tumor cells with low expression levels of GPX4, DHODH enhances susceptibility to ferroptosis by utilizing CoQ_10_ to eliminate intra-mitochondrial ROS. Further studies have shown that the DHODH inhibitor Brequinar induces ferroptosis in cells with low GPX4 expression and also amplifies ferroptosis stimulation in cells with high GPX4 expression (Mao et al., 2021). Thus, DHODH is identified as a novel defense mechanism against ferroptosis in mitochondria. Although both DHODH and FSP1 are non-GSH-dependent anti-ferroptosis molecules, the precise mechanism by which Brequinar induces ferroptosis in cells remains unclear, particularly since Brequinar has also been shown to target FSP1 to sensitize ferroptosis (Mishima et al., 2023; Mao et al., 2023). Furthermore, the common characteristic of DHODH and FSP1 is their reliance on CoQ_10_; however, whether additional interactions are involved is still unknown.

### Ferroptosis in spinal cord injury

Spinal cord injury (SCI) is an acute traumatic condition of the central nervous system that results in significant motor, sensory, and autonomic dysfunction. The detailed mechanisms underlying SCI remain unclear. Given the irreversibility of the primary injury, treatments aimed at mitigating secondary injury are the primary strategies, with a focus on preventing neuronal cell death being a key area of research. Various forms of neuronal cell death can occur following SCI, including autophagy, apoptosis, pyroptosis, necroptosis, and ferroptosis. Notably, ferroptosis has been shown to play a role in the progression of SCI, and targeting ferroptosis may help reduce oxidative damage associated with this condition (Feng et al., 2021).

The earliest study reported that spinal cord injury (SCI) is associated with pathogenic changes indicative of a ferroptosis phenotype. The ferroptosis inhibitor SRS 16-86 was found to attenuate ferroptosis and promote functional recovery in contusion-related SCI (Zhang et al., 2019). This is the first report to reveal the involvement of ferroptosis in SCI. SRS 16-86, as a ferroptosis inhibitor, elevates the expression of GPX4, glutathione (GSH), and xCT, while also downregulating 4-hydroxynonenal (4-HNE) to mitigate ferroptosis. Additionally, it decreases inflammatory biomarkers such as Interleukin-1 beta (IL-1β), Tumor Necrosis Factor-alpha (TNF-α), and Intercellular Adhesion Molecule 1 (ICAM-1). Furthermore, deferoxamine, an iron chelator, has been demonstrated to promote spinal cord repair by inhibiting ferroptosis in rat SCI models and protecting against erastin-induced ferroptosis in primary cortical neurons (Yao et al., 2019; Zhang et al., 2020). Other ferroptosis inhibitors, such as liproxstatin-1 and ferrostatin-1, have also been shown to alleviate SCI by reducing ferroptosis (Fan et al., 2021; Ge et al., 2022). Meanwhile, several endogenous factors, including Growth Differentiation Factor 15 (GDF15) (Xia et al., 2022), Fibroblast Growth Factor 21 (FGF21) (Gu et al., 2024; Xu et al., 2023a), cathepsin B (CTSB) (Xu et al., 2023b), and synoviolin 1 (SYVN1) (Guo et al., 2023) have been corroborated to play critical roles in the occurrence of ferroptosis in SCI. Several novel biomaterials have also been designed to target reactive oxygen species (ROS) scavenging and inhibit ferroptosis, thereby ameliorating SCI (Hua et al., 2024; Zhou et al., 2024; Sun et al., 2024). Overall, research on ferroptosis in SCI has emerged in recent years, and the underlying mechanisms still require further investigation. Numerous studies have reported the efficacy of natural products in treating SCI by suppressing ferroptosis; however, a comprehensive summary of relevant key points is lacking. This review provides detailed insights into the studies of natural products in SCI related to ferroptosis, suggesting potential new research directions for the future.

### Natural products inhibit ferroptosis in spinal cord injury

To date, numerous types of natural products have been investigated in relation to the pathophysiology of spinal cord injury (SCI). These treatment strategies primarily aim to promote the survival of neurons by reducing the severity of inflammation and immunological damage. A wide array of therapeutic targets for natural products that contribute to the improvement of SCI has been extensively documented. However, research on ferroptosis in the context of SCI has only emerged in recent years, and the use of natural products to treat SCI through the inhibition of ferroptosis is rarely discussed. Therefore, the following summarizes studies on natural products that mitigate SCI by suppressing ferroptosis (also illustrated in Figure 1; Table 1).

**TABLE 1:** The summary of natural products in the treatment of SCI by mediating ferroptosis inhibition.

Year	Authors	Natural products	*In vitro* studies	*In vivo* studies	Ferroptosis inducers	Ferroptosis inhibitors	Target molecules	Signaling pathways	Brief description
2020	Jianjun Wang et al.	(−)-Epigallocatechin-3-gallate (EGCG)	Cerebellar granule neurons	—	Erastin, H_2_O_2_	—	GPX4	ERK1/2	EGCG modulates PKD1 and inhibits ferroptosis to ameliorate SCI
2020	Huangao Zhou et al.	Proanthocyanidin	—	Mice	—	—	GPX4	Nrf2	Proanthocyanidin promotes functional recovery of SCI via inhibiting ferroptosis
2021	Jie Cheng et al.	Carnosic acid	PC12	—	Erastin	Ferrostatin-1	GPX4	Nrf2	Carnosic acid inhibits ferroptosis via activating Nrf2 to upregulate the GSH-GPX4 axis and downregulate cellular iron levels in PC12 cells
2022	Yifei Zhou et al.	Astragaloside IV	PC12	—	H_2_O_2_, FIN56	—	GPX4	—	Astragaloside IV protects PC12 cells against oxidative injury mediated by H_2_O_2_ via expression of TFEB and the subsequent suppression of ferroptosis
2022	Fangyi Gong et al.	Trehalose	Neuronal cells	Mice	Erastin	—	GPX4	Nrf2	Trehalose activates the Nrf2/HO-1 pathway to inhibit ferroptosis and ferroptosis-related inflammation thereby playing neuroprotective roles
2023	Jinpei Yang et al.	8-gingerol	Hippocampal Neurons (HT22)	Rat	RSL-3	Ferrostatin-1	GPX4	—	Eight-gingerol (8G)-loaded mesoporous polydopamine (M-PDA) protects against SCI by inhibiting ferroptosis
2023	Yeyang Wang et al.	Quercetin	Oligodendrocyte progenitor cells	Mice	Erastin	—	GPX4	Id2/transferrin	Quercetin prevents the ferroptosis of primary oligodendrocyte progenitor cells by inhibiting the Id2/transferrin pathway
2023	Chengtao Ni et al.	Resveratrol	—	Mice	—	—	GPX4	Nrf2	Resveratrol inhibits ferroptosis via activating the Nrf2/GPX4 pathway in mice with SCI
2024	Longyu Zhang et al.	Albiflorin	Microglial BV-2	Rat	LPS	—	GPX4	—	Albiflorin Attenuates Neuroinflammation and Improves Functional Recovery After SCI Through Regulating LSD1-Mediated Microglial Activation and Ferroptosis
2024	Rui Zhu et al.	Alpha-tocopherol	PC12	Rat	H_2_O_2_	—	ALOX15	—	Alpha-tocopherol inhibits ferroptosis and promotes neural function recovery in rats with SCI via downregulating Alox15
2024	Wenyuan Shen et al.	Celastrol	Precursor oligodendrocyte OLN-93	Rat	Erastin	Ferrostatin-1	GPX4	Nrf2	Celastrol inhibits oligodendrocyte and neuron ferroptosis to promote SCI recovery
2024	Xin Hong et al.	Hispolon	Hippocampal cell HT22	—	Erastin	—	GPX4	Nrf2	Hispolon inhibits neuronal ferroptosis by promoting the expression of Nrf-2
2024	Jian Huang et al.	Dendrobium Nobile Polysaccharide (DNP)	—	Rat	—	—	GPX4	—	Dendrobium Nobile Polysaccharide facilitates post-injury recovery in SCI rats via the inhibition of ferroptosis
2024	Arman Vahabi et al.	Silibinin	—	Rat	—	—	GPX4	—	Silibinin promotes healing in SCI through the inhibition of ferroptosis
2024	Gang Liu et al.	Tetramethylpyrazine	—	Rat	RSL-3	—	GPX4	—	Tetramethylpyrazine alleviates ferroptosis and promotes functional recovery in spinal cord injury by regulating GPX4/ACSL4

EGCG, (−)-Epigallocatechin-3-gallate; H_2_O_2_, hydrogen peroxide; GPX4, Glutathione Peroxidase 4; PKD1, Protein Kinase D1; SCI, spinal cord injury; Nrf2, Nuclear factor-erythroid 2 Related Factor 2; TFEB, Transcription Factor EB; Id2, Inhibitor of DNA, Binding 2; LPS, lipopolysaccharide; LSD1, Lysine-Specific Demethylase 1; ALOX15, Arachidonic acid 15-lipoxygenase; ACSL4, Acyl-CoA, Synthetase Long Chain Family Member 4.

### (−)-Epigallocatechin-3-gallate (EGCG)

Previous studies have identified that the abundant catechins found in green tea are beneficial for the nervous system. (−)-Epigallocatechin-3-gallate (EGCG) is one of the primary catechins in *Camellia sinensis* (green tea). According to relevant reports, EGCG plays a therapeutic role in anti-inflammation and reduces oxidative damage. Mechanistically, EGCG primarily inhibits the activation of the TNF-α-mediated nuclear factor-kappa B (NF-κB) pathway, thereby mitigating the severity of inflammation, and activates the Nrf2/Heme oxygenase-1 (HO-1) pathway to alleviate oxidative stress (Khalatbary and Ahmadvand, 2011; Wang et al., 2022). An early study demonstrated that EGCG significantly decreased malondialdehyde (MDA) levels and altered the ratio of B-cell lymphoma-2 (Bcl2) to Bcl2-associated X (Bax), thereby protecting the spinal cord from secondary injury in a rat model (Khalatbary et al., 2010). Furthermore, intraperitoneal injection of EGCG at a dose of 50 mg/kg attenuates the expression of inflammatory cytokines, including TNF-α, IL-1β, nitrotyrosine, inducible nitric oxide synthase (iNOS), cyclooxygenase-2 (COX-2), and poly (ADP-ribose) polymerase (PARP) (Khalatbary and Ahmadvand, 2011). It can be concluded that EGCG is effective in protecting against spinal cord injury (SCI) by inhibiting inflammatory reactions. Further studies have also verified that EGCG reduces spinal cord edema after SCI by downregulating the protein expression levels of aquaporin-4 (AQP4) and glial fibrillary acidic protein (GFAP) (Ge et al., 2013). Regarding ferroptosis in SCI, EGCG modulates PKD1 and inhibits ferroptosis by enhancing GPX4 through the ERK1/2 signaling pathway, thereby ameliorating SCI in rats (Wang et al., 2020).

### Proanthocyanidin

Proanthocyanidins (PACs), also known as condensed tannins, are a class of natural polyphenolic compounds that are widely present in plants. PACs extracted from grape seeds exhibit antioxidant properties by neutralizing free radicals, which play significant roles in various biological processes (Bagchi et al., 2014). Moreover, intraperitoneal administration of the proanthocyanidins-rich fraction (PRF) obtained from *Croton celtidifolius* bark effectively ameliorates SCI and glutamatergic excitotoxicity in rat models (Assis et al., 2014). In hydrogen peroxide (H_2_O_2_)-treated adrenal pheochromocytoma PC12 cells, the addition of PAC inhibits oxidative stress and mitochondrial apoptosis by activating the PI3K/AKT pathway (He et al., 2021). Moreover, PAC was also reported to protect against iron overload-induced neuronal apoptosis by sustaining the balance of mineral elements, reducing oxidative stress, and inhibiting apoptosis (Yun et al., 2020). These two studies collectively highlight the role of PAC in neuroprotection through its anti-ferroptosis effects. Direct evidence indicates that PACs exert protective effects on SCI repair by disrupting GSH/GPX4 depletion, preventing iron accumulation, and mitigating lipid peroxidation in adult female mice (Zhou et al., 2020).

### Carnosic acid

Carnosic acid (CA), primarily derived from *Rosmarinus officinalis* and *Salvia officinalis* is proposed to gain the anti-oxidative stress, anti-inflammatory, and anti-carcinogenic properties (Bahri et al., 2016). CA is an ortho-dihydroquinone compound that becomes electrophilic upon reaction with free radicals. Notably, CA acts as a specific Nrf2/ARE activator by binding to Keap1, thereby activating the Nrf2 signaling pathway, which exhibits anti-oxidative effects (Yang et al., 2017). CA is recognized for its potent neuroprotective efficacy, particularly in modulating glutathione (GSH) synthesis and downregulating neurotrophin levels (Maruoka et al., 2011). Furthermore, CA demonstrates analgesic effects through the activation of Sirtuin1 and the inhibition of p66shc (Chen et al., 2016). The application of CA in the treatment of spinal cord injury (SCI) has been explored. CA mitigates Erastin-induced ferroptosis in PC12 cells by regulating GSH synthesis and metabolism, as well as cellular iron homeostasis, effectively reversing elevated levels of malondialdehyde (MDA), iron, and reactive oxygen species (ROS), while increasing GSH levels. The inhibitory effect of CA on ferroptosis is mediated by the activation of the Nrf2 signaling pathway (Cheng et al., 2021). However, the therapeutic efficacy of CA in SCI animal models remains to be fully elucidated.

### Astragaloside IV

Astragaloside IV (AS-IV) is an extract derived from the traditional Chinese medicine *Astragalus membranaceus*. It has been shown to eliminate toxins, promote tissue regeneration, reduce swelling, and enhance diuresis (Auyeung et al., 2016). Additionally, AS-IV exhibits potent antioxidant properties by targeting free radicals and reducing lipid peroxidation (You et al., 2017). It counteracts reactive oxygen species (ROS) production, thereby mitigating oxidative stress damage. Recent studies have confirmed that AS-IV plays neuroprotective pharmacological roles by inhibiting inflammation and oxidation (Costa et al., 2019). In the context of spinal cord injury (SCI), AS-IV-mediated suppression of mTORC1 has been demonstrated to attenuate both microglial inflammatory responses and neuronal apoptosis, while promoting functional recovery (Lin et al., 2020). Research on the anti-ferroptosis role of AS-IV in SCI indicates that AS-IV alleviates H2O2-induced damage in PC12 cells by promoting the expression of the transcription factor EB (TFEB) and subsequently suppressing ferroptosis (Zhou et al., 2022). However, further studies using ferroptosis SCI animal models are needed to explore the role of AS-IV more comprehensively.

### Trehalose

Trehalose is a disaccharide that is widely distributed in bacteria, fungi, plants, and invertebrates (Takahashi et al., 2014). It has been shown to inhibit inflammatory responses and oxidative stress. Trehalose exhibits cytoprotective effects under various stress conditions. Previous studies have reported that trehalose protects *Drosophila* and mammalian cells from anoxic stress (Chen et al., 2002). Additionally, several studies have revealed the neuroprotective role of trehalose in neurological diseases, including Alzheimer’s disease (AD), Huntington’s disease (HD), and amyotrophic lateral sclerosis (ALS) (Tanaka et al., 2004; Du et al., 2013). The mechanisms by which trehalose exerts its therapeutic effects in spinal cord injury (SCI) remain enigmatic. Trehalose has been found to alleviate SCI by downregulating matrix metalloproteinase-2 (MMP-2) and MMP-9 (Mirzaie et al., 2018). Collectively, it has been reported that trehalose protects against SCI through the regulation of inflammation, inhibition of oxidative stress, attenuation of apoptosis, and promotion of autophagy via mTOR-independent activation (Zhou et al., 2021; Nazari-Robati et al., 2019; Nasouti et al., 2019). Notably, the neuroprotective effects of trehalose following SCI are also associated with the activation of the Nrf2/HO-1 pathway, which inhibits ferroptosis and ferroptosis-related inflammation (Gong et al., 2022).

### Gingerol

Ginger is a highly valuable economic crop with significant potential for development in the pharmaceutical, food, and spice industries. However, there are relatively few reports on gingerol and its effects. Gingerol, primarily extracted from ginger, is a phenolic compound known for its potent antioxidant and anti-inflammatory properties (Dugasani et al., 2010). Previous studies have demonstrated that 6-gingerol exerts a therapeutic effect on neuroinflammation associated with sciatic nerve damage by decreasing inflammatory cytokines such as TNF-α, IL-1β, and IL-18 (Özdemir et al., 2023). Additionally, gingerol has been shown to protect against diabetes mellitus by inhibiting ferroptosis through the enhancement of the Nrf2/HO-1 pathway and by reducing inflammation via the suppression of inflammatory cytokines, including IL-1β, IL-6, and TNF-α (Wu et al., 2022). In the context of spinal cord injury (SCI) treatment, 8-gingerol (8G)-loaded mesoporous polydopamine (M-PDA) significantly reduced the local injury area and mitigated axonal and myelin loss, thereby improving neurological and motor recovery in rats (Yang et al., 2023). Mechanistically, 8G-loaded M-PDA appears to diminish lipid peroxidation and inhibit secondary SCI by suppressing ferroptosis and inflammation.

### Quercetin

Quercetin is a redox-active flavonoid that serves as a critical component in traditional Chinese medicine. It offers numerous benefits, including antioxidative properties through the scavenging of free radicals (Nabavi et al., 2012). Various studies have elucidated the therapeutic roles of quercetin in spinal cord injury (SCI) through different mechanisms. For instance, quercetin has been shown to attenuate the recruitment of neutrophils and reduce myeloperoxidase (MPO) release at the site of SCI in animal models (Schültke et al., 2010). Furthermore, quercetin has been demonstrated to mitigate monosodium glutamate-induced excitotoxicity in spinal cord motoneurons by inhibiting the p38-MAPK signaling pathway (Firgany and Sarhan, 2020). Recent findings indicate that quercetin decreases the area of injury and significantly downregulates the expression of Id2 and transferrin, while upregulating the expression of GPX4 (Wang et al., 2023). Overall, this study suggests that quercetin prevents ferroptosis in oligodendrocyte progenitor cells by inhibiting the Id2/transferrin pathway, which may propose a potential therapeutic strategy for the inhibition of ferroptosis in SCI.

### Resveratrol

Resveratrol is a polyphenolic compound predominantly extracted from grape skins, peanuts, and various medicinal plants. Research has demonstrated that resveratrol influences both the pathological and physiological processes associated with inflammation and injury in the body. As a natural plant component with potent biological activity, resveratrol exhibits a range of effects, including tumor inhibition, anti-infection properties, anti-inflammatory actions, and protection of the cardiovascular and cerebrovascular systems (Song et al., 2024). It has gradually emerged as a prominent focus in research areas such as cancer. Currently, numerous studies are dedicated to elucidating the underlying mechanisms by which resveratrol aids in the treatment of spinal cord injury (SCI). A recent report confirms that resveratrol plays a significant role in antioxidation and promotes neuronal recovery (Tang et al., 2024). Research conducted by Ni et al. has directly shown that resveratrol enhances motor function following SCI (Ni et al., 2023). Additionally, resveratrol inhibits the expression of ferroptosis-related genes, prevents iron accumulation, and improves mitochondrial morphology as observed through transmission electron microscopy (TEM). Mechanistically, resveratrol has been shown to inhibit ferroptosis via the Nrf2/GPX4 pathway, thereby alleviating the effects of SCI.

### Albiflorin

Albiflorin, a monoterpenoid glycoside primarily derived from the roots of *Paeonia lactiflora*, is a commonly used Chinese herbal medicine (Li et al., 2024b). Previous studies have demonstrated that the main functions of albiflorin include the inhibition of oxidative injury, anti-inflammatory effects, and immune modulation (Ou et al., 2024). Additionally, it has been reported that albiflorin has the potential to reduce apoptosis and necrosis while sustaining cellular mitochondrial functions (Suh et al., 2013). Early research indicated that albiflorin possesses analgesic properties that significantly alleviate neuropathic pain in rats with chronic constriction injury by suppressing the overexpression of phosphorylated c-Jun N-terminal kinases (p-JNK) in astrocytes and decreasing the levels of the chemokine CXCL1 in the spinal cord (Zhou et al., 2016). These findings suggest that albiflorin may serve as a promising target for therapeutic intervention in spinal cord injury (SCI). Existing evidence shows that albiflorin effectively alleviates motor neuron dysfunction and neuronal cell death by reducing oxidative stress, promoting glutathione biosynthesis, and activating the Nrf2/HO-1 signaling pathway in rat models (Fang et al., 2023b). Further investigations have indicated that albiflorin reduces microglial activation and ferroptosis, thereby attenuating neuroinflammation and enhancing functional recovery following SCI by downregulating LSD1 (Zhang et al., 2024).

### Alpha-tocopherol

Alpha-tocopherol, also known as Vitamin E (Vit E), is widely recognized as an effective natural antioxidant that scavenges lipophilic free radicals (Tian et al., 2024). Additionally, alpha-tocopherol plays a crucial role in modulating immune responses and exhibits anti-inflammatory properties, while also providing protection against oxidative damage. Numerous studies have reported that alpha-tocopherol exerts protective effects in neurological disorders such as epilepsy, Alzheimer’s disease, and Parkinson’s disease, and it has been shown to enhance the recovery of motor function in animal models of spinal cord injury (SCI) (Browne et al., 2019; Morsy et al., 2010). The recovery of motor neurons following SCI mediated by alpha-tocopherol occurs through complex mechanisms. Furthermore, recent studies have demonstrated that alpha-tocopherol mitigates lipid peroxidation by inhibiting RSL-3-induced ferroptosis (Hu et al., 2021). Both cells and tissues can utilize alpha-tocopherol, which is reduced by FSP1, to protect against severe lipid peroxidation and subsequent ferroptosis (Bersuker et al., 2019; Doll et al., 2019). Indeed, alpha-tocopherol has been shown to inhibit ferroptosis by reducing reactive oxygen species (ROS) accumulation, iron overload, lipid peroxidation, and mitochondrial dysfunction, thereby promoting neural function recovery in rats with SCI through the downregulation of ALOX15 (Zhu et al., 2024).

### Celastrol

Celastrol, also known as phatosporine, is a significant active compound derived from the natural plant *Tripterygium wilfordii* (Liu et al., 2015). Research has demonstrated that celastrol possesses numerous beneficial biological effects, including anti-inflammatory, antioxidant, anticancer properties, and the promotion of weight loss (Liu et al., 2015; Kannaiyan et al., 2011; Li et al., 2015). In the context of treating neurological disorders, celastrol has been identified as a potent therapeutic agent for Alzheimer’s disease, multiple sclerosis, Parkinson’s disease, cerebral ischemia, amyotrophic lateral sclerosis, and nervous system tumors (Bai et al., 2021). Recently, the role of celastrol in promoting recovery from spinal cord injury (SCI), primarily through its anti-inflammatory and antioxidant effects, has also been highlighted (Dai et al., 2019; Li et al., 2023). Furthermore, a recent study has shown that celastrol inhibits ferroptosis by upregulating the Nrf2-xCT-GPX4 axis and reducing the production of lipid reactive oxygen species (ROS), thereby enhancing the survival of both neurons and oligodendrocytes and improving functional recovery following SCI (Shen et al., 2024).

### Hispolon

Hispolon is a naturally occurring polyphenol that can be isolated from *Phellinus linteus*. To date, hispolon has been shown to play critical roles in the treatment of cancer, diabetes mellitus, and viral infections (Sarfraz et al., 2020). Recent studies have also discovered that hispolon protects cells and tissues from oxidative stress and inflammation (Lee et al., 2022). The toxicity and anti-genotoxic effects of hispolon in modulating the cellular redox state have been reported (Chethna et al., 2018). Hispolon mitigates oxidative damage-induced cell death in PC12 cells by activating Nrf2-regulated antioxidant genes in a dose-dependent manner, positioning it as an effective activator of Nrf2 and a promising candidate for the treatment of neurodegenerative diseases (Peng et al., 2022). A newly published study demonstrated that hispolon can enhance the expression of Nrf2 and inhibit the occurrence of neuronal ferroptosis induced by erastin, suggesting a potential therapeutic strategy for treating spinal cord injury (SCI) (Hong et al., 2024).

### Dendrobium nobile polysaccharide

Dendrobium nobile polysaccharide (DNP), also known as Jinchaishihu, is a compound primarily derived from *Dendrobium nobile* (Hsu et al., 2024). DNP is a traditional Chinese medicine that serves multiple functions, including antioxidant activity, inhibition of lipid peroxidation, suppression of inflammatory responses, and immune modulation (Wang et al., 2018). Early studies have reported the neuroprotective effects and anti-ferroptosis mechanisms of DNP in the context of vascular dementia (Ming et al., 2023). Treatment with DNP resulted in the upregulation of glutathione (GSH), cystine/glutamate transporter (xCT), and glutathione peroxidase 4 (GPX4) expressions in the hippocampus. Furthermore, synapses remained relatively intact, with an increase in synaptic vesicles and a significant elongation of the synaptic active zone observed following DNP administration. Overall, DNP mitigates ferroptosis and enhances cognitive function in cases of vascular dementia. In rats with spinal cord injury (SCI), DNP promotes neural recovery and inhibits ferroptosis by upregulating the expression of xCT, GPX4, and GSH (Huang et al., 2024).

### Silibinin

Silymarin is derived from the milk thistle plant, *Silybum marianum*, and has been used worldwide for the long-term treatment of liver diseases, including hepatitis, alcoholic fatty liver, nonalcoholic fatty liver disease, and drug-induced liver injury (Soleimani et al., 2019). Silibinin, also known as silybin, is the primary active component of silymarin, comprising 60%–70% of its content (Bijak, 2017). Research has demonstrated that silibinin plays critical roles in antioxidant, anti-inflammatory, and anti-fibrotic activities (Duan et al., 2023). Furthermore, numerous studies indicate that silibinin protects neuronal cells from damage, including oxidative stress and inflammatory responses (Wadhwa et al., 2022; Tsai et al., 2010). Additionally, silibinin has been shown to suppress ferroptosis, which may help ameliorate tissue injuries, suggesting that it could serve as a potential therapeutic agent for ferroptosis-related diseases (Duan et al., 2023). In the context of ferroptosis-mediated cell death following spinal cord injury (SCI), silibinin emerges as a promising therapeutic candidate by influencing iron metabolism and lipid peroxidation associated with ferroptosis (Vahabi et al., 2024).

### Tetramethylpyrazine

Tetramethylpyrazine (TMP) is a monomer derived from the traditional Chinese herbal plant *Ligusticum wallichii Franchat*, commonly known as Chuanxiong. This compound was first documented during the Tang dynasty in China (Zhuang et al., 2020). TMP has been incorporated into various clinical drugs and is widely utilized in clinical practice. Previous studies have validated that TMP possesses numerous beneficial effects, including the suppression of inflammation, scavenging of reactive oxygen species (ROS), inhibition of lipid peroxidation, protection of mitochondria, and enhancement of microcirculation (Wang et al., 2024b). Additionally, findings have corroborated that TMP protects against neuronal damage in Parkinson’s disease by scavenging free oxidative radicals (Lu et al., 2014). Further mechanisms elucidating the neuroprotective roles of TMP have also been identified (Chen et al., 2020). In the context of spinal cord injury (SCI), TMP has been shown to alleviate ferroptosis by regulating the expression of GPX4 and ACSL4, thereby promoting functional recovery in SCI (Liu et al., 2024b).

## Conclusions and prospectives

The mechanisms underlying spinal cord injury (SCI) remain enigmatic and require further elucidation. Cell death is a critical event in the acute pathological process of SCI. The involvement of ferroptosis in SCI presents a potential avenue for treatment. It is possible that local bleeding during the acute phase of SCI leads to a rapid increase in iron levels, and this iron overload further exacerbates the accumulation of reactive oxygen species (ROS), thereby inducing neuronal ferroptosis. Research has shown that ferroptosis inhibitors can effectively rescue spinal cord neurons by preventing ferroptosis, as well as alleviating inflammatory biomarkers, astrocyte activation, iron accumulation, and ROS levels. This offers new hope for the rehabilitation of SCI patients.

Currently, three well-known cellular enzymatic antioxidants including GPX4, FSP1, and DHODH are elucidated to be involved in ferroptosis defense. GPX4, which functions as the main role of ROS scavenging, is regarded as the critical gatekeeper for ferroptosis. Numerous studies identified that natural products with anti-oxidant roles in treating SCI are targeting GPX4 to inhibit ferroptosis. However, the FSP1- and DHODH-related mechanisms are rarely unveiled during these molecules involved in SCI. In addition, the unknown existence of interactions between ferroptosis and SCI still needs to be elucidated. Studies focusing on natural products and SCI might reveal novel mechanisms in ferroptosis. Downstream mechanisms for these natural products mediating anti-ferroptosis efficacy are mainly centered at the Nrf2-related anti-oxidative stress signaling pathway. Numerous molecular pathways involved in ferroptosis have been corroborated.

In this review, several natural products have been used in the treatment of SCI for the downregulation of inflammation, alleviation of edema, and promotion of neural recovery. Relevant mechanisms of these natural products have been widely reported previously. However, recent findings also provide evidence about their roles in anti-ferroptosis among using in SCI. These novel elaborations reveal the complicated mechanisms of natural products when utilized on humans. Also, more additional natural products could be focused on their applications in SCI treatment. Nevertheless, natural products with anti-ferroptosis roles are solely transferred to the clinical trials though indeed abundant authentications supporting their availability. The attempt at clinical trials about natural products might be regarded as a key focus for future studies.
